# Supplementary material for: Microvascular Cortical Dynamics in Minimal Invasive Deep-Seated Brain Tumour Surgery
Source: Cancers (Basel). 2025 Apr 22;17(9):1392. doi: 10.3390/cancers17091392 (PMC12070978; doi:10.3390/cancers17091392)
Supplement: Supplementary file 1 [file cancers-17-01392-s001.zip › Supplementary Material S1.pdf]

Adjusted Analysis for Quantitative Flow Metrics Pre-Cannulation and Focal Neurological Deficit

	Coef.	95%CI	P value
Delay	-0.07±0.05	[-0.17 – 0.03]	0.145
MAP at cannulation	-0.06±0.02	[-0.09 – -0.02]	0.002
Distance Tumour-to-Surface	-0.47±0.17	[-0.81 – -0.13]	0.007
Initial Tumour Volume	0.02±0.01	[0.01 – 0.04]	0.010
Speed	0.01±0.003	[0.004 – 0.02]	0.001
MAP at cannulation	-0.06±0.02	[-0.10 – -0.02]	0.003
Distance Tumour-to-Surface	-0.82±0.22	[-1.25 – -0.38]	<0.0001
Initial Tumour Volume	0.03±0.01	[0.01 – 0.04]	0.004
Time to Peak	-0.13±0.04	[-0.20 – -0.06]	<0.0001
MAP at cannulation	-0.06±0.02	[-0.10 – -0.02]	0.002
Distance Tumour-to-Surface	-0.61±0.20	[-1.00 – -0.22]	0.002
Initial Tumour Volume	0.02±0.01	[0.003 – 0.04]	0.023
Rise in Time	-0.29±0.07	[-0.43 – -0.15]	<0.0001
MAP at cannulation	-0.06±0.02	[-0.10 – -0.02]	0.003
Distance Tumour-to-Surface	-0.60±0.21	[-1.12 – -0.28]	0.001
Initial Tumour Volume	0.02±0.01	[0.003 – 0.04]	0.028
Cerebral Blood Flow Index	0.17±0.09	[-0.01 – 0.35]	0.058
MAP at cannulation	-0.07±0.03	[-0.12 – -0.02]	0.007
Distance Tumour-to-Surface	-0.80±0.29	[-1.37 – -0.24]	0.005
Initial Tumour Volume	0.06±0.02	[0.20 – 0.10]	0.005

Adjusted Analysis for Quantitative Flow Metrics Post-Decannulation and Overall Neurology Outcome

	Coef.	95%CI	P value
Delay	0.06±0.06	[-0.06 – 0.17]	0.284
MAP at decannulation	0.02±0.01	[-0.0005 –0.05]	0.055
Postoperative Volume of Ischemia	-0.17±0.08	[-0.33 – -0.01]	<u>0.038</u>
Residual Tumour Volume	0.02±0.02	[-0.01 – 0.05]	0.267
Speed	-0.003±0.004	[-0.01 – 0.005]	0.461
MAP at decannulation	0.02±0.01	[-0.002 – 0.05]	0.072
Postoperative Volume of Ischemia	-0.18±0.08	[-0.34 – -0.02]	<u>0.029</u>
Residual Tumour Volume	0.02±0.02	[-0.01 – 0.05]	0.252
Time to Peak	0.09±0.04	[0.01 – 0.17]	<u>0.022</u>
MAP at decannulation	0.02±0.01	[-0.003 – 0.05]	0.088
Postoperative Volume of Ischemia	-0.23±0.09	[-0.40 – -0.06]	<u>0.008</u>
Residual Tumour Volume	0.02±0.02	[-0.01 – 0.05]	0.298
Rise in Time	0.16±0.06	[0.04 – 0.27]	<u>0.007</u>
MAP at decannulation	0.02±0.01	[-0.001 – 0.04]	0.153
Postoperative Volume of Ischemia	-0.26±0.09	[-0.43 – -0.08]	<u>0.004</u>
Residual Tumour Volume	0.02±0.02	[-0.01 – 0.05]	0.170
Cerebral Blood Flow Index	-0.19±0.07	[-0.33 – -0.06]	<u>0.006</u>
MAP at decannulation	0.02±0.01	[-0.001 – 0.05]	0.149
Postoperative Volume of Ischemia	-0.34±0.010	[-0.53 – -0.14]	<u>0.001</u>
Residual Tumour Volume	0.02±0.01	[-0.01 – 0.05]	0.186

Adjusted Analysis for Quantitative Flow Metrics Comparing the Difference between Post-Decannulation and Pre-Cannulation and Overall Neurology Outcome

	Coef.	95%CI	P value
Delay	-0.0001±0.0001	[-0.0002 – 0.0001]	0.524
MAP at cannulation	0.005±0.0004	[-0.003 – 0.14]	0.206
MAP at decannulation	0.007±0.003	[-0.0001 – 0.014]	0.074
Initial Tumour Volume	0.007±0.003	[0.002 – 0.014]	0.012
Residual Tumour Volume	-0.004±0.004	[-0.014 – 0.005]	0.364
Distance Tumour-to-Surface	0.02±0.04	[-0.06 – 0.10]	0.577
Postoperative Volume of Ischemia	-0.010±0.003	[-0.016 - -0.004]	0.001
Speed	-0.003±0.0006	[-0.004 – -0.002]	<0.0001
MAP at cannulation	0.006±0.0004	[-0.002 – 0.134]	0.149
MAP at decannulation	0.006±0.003	[-0.0007 – 0.012]	0.080
Initial Tumour Volume	0.006±0.003	[0.0002 – 0.011]	0.049
Residual Tumour Volume	-0.0001±0.004	[-0.009 – 0.009]	0.981
Distance Tumour-to-Surface	0.04±0.04	[-0.03 – 0.11]	0.271
Postoperative Volume of Ischemia	-0.010±0.003	[-0.013 - -0.002]	0.004
Time to Peak	0.0005±0.0004	[-0.003 – 0.001]	0.241
MAP at cannulation	0.004±0.0004	[-0.04 – 0.13]	0.260
MAP at decannulation	0.007±0.003	[0.0002 – 0.014]	0.042
Initial Tumour Volume	0.007±0.003	[0.001 – 0.013]	0.029
Residual Tumour Volume	-0.003±0.004	[-0.013 – 0.006]	0.496
Distance Tumour-to-Surface	0.02±0.04	[-0.06 – 0.10]	0.574
Postoperative Volume of Ischemia	-0.009±0.003	[-0.015 - -0.003]	0.003
Rise in Time	0.0003±0.0002	[-0.0002 – 0.0008]	0.262
MAP at cannulation	0.004±0.0004	[-0.04 – 0.13]	0.269
MAP at decannulation	0.007±0.004	[0.0003 – 0.014]	0.039
Initial Tumour Volume	0.007±0.003	[0.008 – 0.013]	0.025
Residual Tumour Volume	-0.003±0.005	[-0.012 – 0.006]	0.481
Distance Tumour-to-Surface	0.02±0.04	[-0.06 – 0.10]	0.579
Postoperative Volume of Ischemia	-0.009±0.003	[-0.015 - -0.003]	0.003
Cerebral Blood Flow Index	-0.003±0.0008	[-0.004 – -0.001]	0.001
MAP at cannulation	-0.004±0.0004	[-0.014 – 0.005]	0.383
MAP at decannulation	0.006±0.004	[0.0003 – 0.014]	0.139
Initial Tumour Volume	0.007±0.004	[-0.006 – 0.015]	0.068
Residual Tumour Volume	-0.02±0.05	[-0.018 – 0.006]	0.305
Distance Tumour-to-Surface	-0.02±0.04	[-0.08 – 0.11]	0.781
Postoperative Volume of Ischemia	0.79±0.62	[-0.105 - -0.003]	0.038
