# Supplementary material for: Microvascular Cortical Dynamics in Minimal Invasive Deep-Seated Brain Tumour Surgery
Source: Cancers (Basel). 2025 Apr 22;17(9):1392. doi: 10.3390/cancers17091392 (PMC12070978; doi:10.3390/cancers17091392)
Supplement: Supplementary file 1 [file cancers-17-01392-s001.zip › Supplementary Material S2.pdf]

Adjusted Analysis for Quantitative Flow Metrics Pre-Cannulation and Focal Neurological Deficit

	Coef.	95%CI	P value
Delay	-0.01±0.10	[-0.21 – 0.18]	0.872
MAP	-0.21±0.12	[-0.44 – 0.02]	0.079
Distance Tumour-to-Surface	-0.92±0.50	[-1.90 – 0.05]	0.063
Tumour Volume	0.01±0.02	[-0.03 – 0.04]	0.639
Speed	0.01±0.01	[-0.002 – 0.02]	0.097
MAP	-0.19±0.12	[-0.42 – 0.05]	0.120
Distance Tumour-to-Surface	-1.14±0.57	[-2.25 – 0.02]	0.046
Tumour Volume	0.01±0.02	[-0.02 – 0.04]	0.562
Time to Peak	-0.13±0.08	[-0.28 – 0.03]	0.103
MAP	-0.17±0.12	[-0.39 – 0.04]	0.115
Distance Tumour-to-Surface	-0.89±0.46	[-1.80 – 0.02]	0.054
Tumour Volume	0.004±0.02	[-0.03 – 0.03]	0.801
Rise	-0.22±0.15	[-0.51 – 0.07]	0.136
MAP	-0.19±0.12	[-0.42 – 0.04]	0.115
Distance Tumour-to-Surface	-1.03±0.53	[-2.06 – 0.003]	0.051
Tumour Volume	0.004±0.02	[-0.03 – 0.03]	0.799
Cerebral Blood Flow Index	0.26±0.23	[-0.19 – 0.70]	0.255
MAP	-0.10±0.14	[-0.38 – 0.18]	0.490
Distance Tumour-to-Surface	-1.19±0.71	[-2.57 – 0.19]	0.092
Tumour Volume	0.03±0.04	[-0.04 – 0.11]	0.363

Adjusted Analysis for Quantitative Flow Metrics Post-Decannulation and Overall Neurology Outcome

	Coef.	95%CI	P value
Delay	0.03±0.04	[-0.05 – 0.10]	0.450
MAP at decannulation	0.02±0.02	[-0.30 –0.07]	0.418
Postoperative Volume of Ischemia	-0.01±0.01	[-0.02 – 0.004]	0.204
Residual Tumour Volume	-0.01±0.01	[-0.02 – 0.02]	0.479
Speed	-0.001±0.003	[-0.007 – 0.006]	0.877
MAP at decannulation	0.02±0.02	[-0.30 –0.07]	0.495
Postoperative Volume of Ischemia	-0.007±0.006	[-0.02 – 0.004]	0.201
Residual Tumour Volume	-0.007±0.01	[-0.02 – 0.03]	0.521
Time to Peak	0.02±0.02	[-0.30 –0.07]	0.439
MAP at decannulation	0.01±0.02	[-0.30 –0.06]	0.552
Postoperative Volume of Ischemia	-0.01±0.01	[-0.02 – 0.003]	0.167
Residual Tumour Volume	0.006±0.01	[-0.01 – 0.03]	0.540
Rise in Time	0.03±0.03	[-0.04 –0.09]	0.368
MAP at decannulation	0.01±0.02	[-0.04 –0.06]	0.585
Postoperative Volume of Ischemia	-0.01±0.01	[-0.02 – 0.004]	0.203
Residual Tumour Volume	0.007±0.01	[-0.01 – 0.03]	0.513
Cerebral Blood Flow Index	-0.04±0.05	[-0.16 –0.07]	0.431
MAP at decannulation	0.01±0.03	[-0.05 –0.07]	0.685
Postoperative Volume of Ischemia	-0.01±0.01	[-0.04 – 0.02]	0.563
Residual Tumour Volume	0.01±0.01	[-0.02 – 0.04]	0.706

Adjusted Analysis for Quantitative Flow Metrics Comparing the Difference between Post-Decannulation and Pre-Cannulation and Overall Neurology Outcome

	Coef.	95%CI	P value
Delay	-0.0004±0.001	[-0.003 – 0.002]	0.752
MAP at cannulation	0.03±0.03	[-0.04 – 0.09]	0.394
MAP at decannulation	0.01±0.03	[-0.04 – 0.07]	0.602
Initial Tumour Volume	0.01±0.01	[0.006 – 0.02]	0.218
Residual Tumour Volume	0.001±0.01	[-0.02 – 0.03]	0.931
Distance Tumour-to-Surface	-0.05±0.10	[-0.25 – 0.15]	0.587
Postoperative Volume of Ischemia	-0.01±0.01	[-0.02 - 0.003]	0.144
Speed	-0.003±0.001	[-0.007 – -0.0004]	0.030
MAP at cannulation	0.03±0.03	[-0.02 – 0.08]	0.274
MAP at decannulation	0.01±0.03	[-0.04 – 0.07]	0.602
Initial Tumour Volume	0.01±0.01	[0.006 – 0.02]	0.218
Residual Tumour Volume	0.001±0.01	[-0.02 – 0.03]	0.931
Distance Tumour-to-Surface	-0.05±0.10	[-0.25 – 0.15]	0.587
Postoperative Volume of Ischemia	-0.01±0.01	[-0.02 - 0.003]	0.144
Time to Peak	0.001±0.001	[-0.001 –-0.004]	0.369
MAP at cannulation	0.02±0.03	[-0.04 – 0.08]	0.421
MAP at decannulation	0.02±0.03	[-0.04 – 0.07]	0.540
Initial Tumour Volume	0.007±0.007	[-0.008 – 0.02]	0.350
Residual Tumour Volume	0.003±0.01	[-0.02 – 0.03]	0.785
Distance Tumour-to-Surface	-0.06±0.09	[-0.25 – 0.13]	0.514
Postoperative Volume of Ischemia	-0.009±0.006	[-0.02 - 0.004]	0.158
Rise in Time	0.004±0.002	[-0.001 – 0.008]	0.120
MAP at cannulation	0.03±0.03	[-0.04 – 0.08]	0.363
MAP at decannulation	0.006±0.03	[-0.05 – 0.06]	0.813
Initial Tumour Volume	0.004±0.007	[-0.01 – 0.02]	0.516
Residual Tumour Volume	0.006±0.01	[-0.02 – 0.03]	0.638
Distance Tumour-to-Surface	-0.07±0.09	[-0.26 – 0.12]	0.434
Postoperative Volume of Ischemia	-0.008±0.006	[-0.02 - 0.005]	0.209
Cerebral Blood Flow Index	-0.006±0.002	[-0.001 – -0.0002]	0.043
MAP at cannulation	-0.002±0.04	[-0.08 – 0.08]	0.960
MAP at decannulation	0.01±0.03	[-0.06 – 0.08]	0.743
Initial Tumour Volume	0.006±0.01	[-0.02 – 0.03]	0.547
Residual Tumour Volume	0.0006±0.02	[-0.04 – 0.04]	0.975
Distance Tumour-to-Surface	-0.04±0.13	[-0.31 – 0.24]	0.788
Postoperative Volume of Ischemia	-0.01±0.01	[-0.04 - 0.01]	0.307

Combined ROI per Patient
